# Anoikis resistance in gastric cancer: a comprehensive review

**DOI:** 10.1038/s41419-025-07860-1

**Published:** 2025-07-15

**Authors:** Teresa D’Amore, Daniele Bravoco, Giuseppina Di Paola, Francesco Albano, Mariarita Brancaccio, Claudia Sabato, Giuseppe Cesta, Cinzia Zolfanelli, Vincenzo Lauciello, Geppino Falco, Pellegrino Mazzone

**Affiliations:** 1 Laboratory of Preclinical and Translational Research, IRCCS-CROB, Referral Cancer Center of Basilicata, Rionero in Vulture, Italy; 2 Biogem Scarl, Istituto di Ricerche Genetiche ‘Gaetano Salvatore’, Ariano Irpino, Italy; 3 Department of Public Health, Federico II University of Naples, Naples, Italy; 4 Department of Biology, University of Naples Federico II, Naples, Italy

**Keywords:** Gastric cancer, Cell death

## Abstract

Gastric cancer (GC) is a predominant malignant neoplasia responsible for cancer death worldwide. Because of the difficulty in early diagnosis as well as its high metastasis rate, GC shows an increasing incidence and poor prognosis. Conventional treatments for GC, such as chemotherapy, radiotherapy, and surgical resection, still fail to achieve curative effects because of drug resistance, a mechanism that leads to a reduction of 5-year survival for GC patients. Anoikis, a particular type of programmed cell death, is activated upon cancer cell detachment from the extracellular matrix, playing a crucial role in antagonizing the progression of several malignant tumors. Because GC cells metastasize mainly in the nearby sites in the peritoneum, a better comprehension of the molecular mechanisms involved in the anchorage-independent growth as well as metastatic spreading is crucial to counteract GC progression. In this context, this review critically examines the molecular mechanisms of anoikis, key pathways and regulatory networks, and the role of anoikis resistance in GC. Furthermore, it summarizes potential therapeutic strategies for targeting anoikis-resistant cells. By collecting and analyzing existing literature, this work aims to bridge gaps in the comprehension of the relation between anoikis resistance and GC pathophysiology, providing novel insights and directions for future research in this field.

## Facts

- Gastric cancer is a leading cause of death worldwide.
- Anoikis is programmed cell death activated upon detachment from the extracellular matrix.
- Anoikis resistance is a hallmark of metastatic cancers.
- Anoikis-related genes are prognostic factors for gastric cancer progression.

## Introduction

Despite recent advancements in the comprehension of the cellular and molecular mechanisms underlying gastric cancer (GC), it still ranks as the fifth leading cause of cancer-related deaths worldwide [1]. Environmental factors such as dietary habits and chronic gastritis due to *Helicobacter pylori* infection, along with genetic mutations, can affect GC incidence around the world with a higher frequency in Eastern countries [2–4]. Currently, the traditional treatments for GC include surgical resection and/or chemotherapy or radiotherapy, although the 5-year survival rate remains extremely low. In addition, the lack of early-stage symptomatology makes this malignancy difficult to diagnose [5]. All differentiated cells grow in proper tissue environments, but when they lose or leave their native environment, apoptosis signal transduction occurs. Anoikis (Greek for “homeless”) is a mechanism of programmed cell death related to apoptosis, sharing similar pathways and components. Particularly, it is activated under specific conditions that lead the cell to detach from the extracellular matrix (ECM). This type of cell death may occur in several diseases, such as tumors, where it is responsible for the detachment and spreading of cancer cells, cardiovascular diseases, in which anoikis triggers the cardiomyocyte detachment in congestive heart failure, and infectious diseases where anoikis disrupts ECM-mediated cell loss of adhesion [6–8]. Cancer cells can evade anoikis, allowing them to survive and spread to distant sites, a phenomenon known as anoikis resistance (AR). This mechanism triggers molecular and biochemical alterations that enhance the invasive and metastatic properties of cancer cells [9]. Additionally, AR contributes to drug resistance and cancer recurrence, posing significant challenges for effective treatment [10, 11]. Recent therapeutic strategies aim to target AR by either promoting anoikis in cancer cells or sensitizing them to programmed cell death [12]. While the general biology of anoikis, AR, and their role in cancer have been previously discussed, GC presents a unique microenvironment and dissemination challenges that necessitate a focused investigation. In this review, we provide a comprehensive analysis of the molecular mechanisms, signaling pathways, and the molecular drivers of AR specifically in GC, incorporating insights from genomic and transcriptomic profiling, including GC-specific signatures (e.g., GC-specific anoikis-related gene and long non-coding RNA signatures). We examine how these molecular alterations correlate with clinical outcomes and immune contexture, and we propose a translational roadmap for targeting AR in GC and improve treatment outcomes. The integration of disease-specific biology with emerging therapeutic strategies provided by this work may be a valuable tool to inform both mechanistic research and the development of precision oncology solutions for GC.

## Molecular pathways involved in anoikis activation

### Activation and regulation of the caspase pathway

**Fig. 1:**
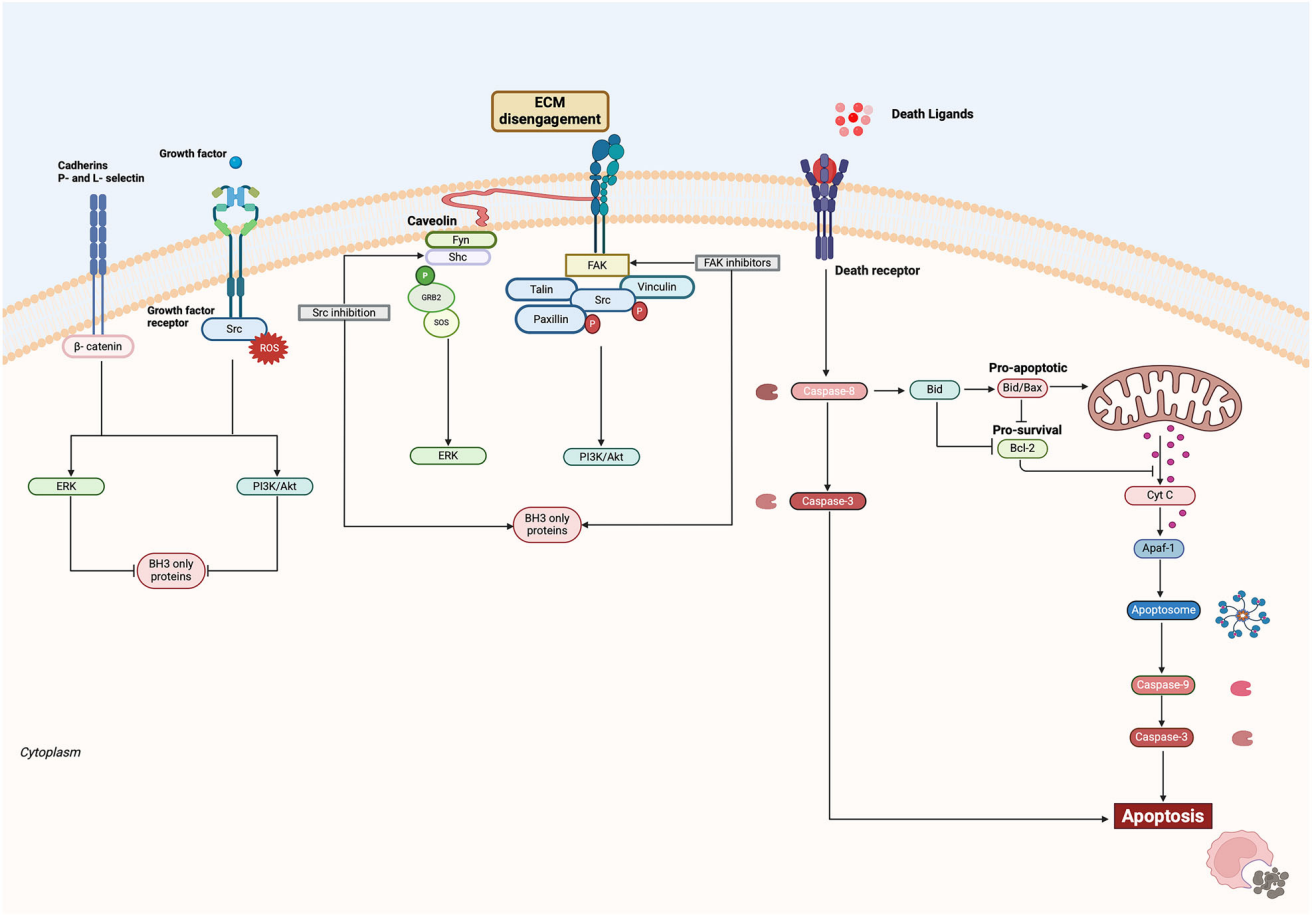
Intrinsic and extrinsic apoptosis pathways. Cell death by anoikis is induced by the activation of the intrinsic or extrinsic apoptosis pathway. The intrinsic pathway is activated by *stimuli* that activate pro-apoptotic proteins, also known as BH-3-only proteins. These proteins inhibit the anti-apoptotic proteins Bcl-2/Bcl-xL, altering the permeability of the outer mitochondrial membrane and determining the release of cytochrome c. Thus, the apoptosome activation triggers caspase 9, an initiator caspase, and consequently, caspase 3, effector caspase. The interplay between IAP proteins and their inhibitors, Smac/Diablo, modulates cell death. In the extrinsic pathway, activated death receptors transduce external signals, ending up in the caspase 8 activation, an initiator caspase, and subsequently caspase 3. In the final stage, neo-formed apoptotic bodies are disrupted by immune cells. Integrin-ECM interactions play a key role in maintaining ECM integrity, which is linked to several key survival factors such as FAK and Src. Growth factor receptors, activated both in a ligand-dependent and -independent manner, cooperate with integrin and laminin to promote cell viability. Cell–cell interactions mediated by cadherins or other cell surface molecules activate signaling pathways similar to those triggered by ECM adhesion.

Being a type of apoptosis, anoikis relies on the activation of caspases, crucial enzymes in the intrinsic and extrinsic apoptotic pathways. In this context, the Bcl-2 family plays a pivotal role in maintaining cell homeostasis. Particularly, this family includes both pro- and antiapoptotic proteins capable of modulating the cell fate upon different *stimuli*. Among the proapoptotic members of this family, Bax and Bak modulate outer mitochondrial membrane (OMM) permeability, allowing the release of cytochrome C, leading to apoptosis activation [13]. On the other hand, Bcl-2 protein exerts its anti-apoptotic activity complexing with Bcl-xL, Bad, and the Apoptosis protease-activating factor-1 [14], preventing the release of cytochrome C, thereby repressing the apoptotic pores formation. Pro-apoptotic proteins, including Noxa, Puma, Bad, Bim, Bmf, and Bik, promote the pro-apoptotic signal induced by genotoxic stress, DNA damage, hypoxia, and other forms of intrinsic cellular damage, such as starvation conditions, and defects in calcium flux, hampering Bcl-2 activity [15–19]. Furthermore, variation of OMM permeability, regulated by the interaction of Bax-Bax or Bax-Bak proteins, leads to the subsequent activation of caspases, thereby initiating the pro-apoptotic cascade that involves initiator caspases, including caspase 9 in the intrinsic pathway and caspase 8 in the extrinsic pathway result into caspase 3 activation [20] (Fig. 1). The extrinsic pathway represents a signal transduction mechanism that is modulated by death factors, including FAS, TNF, and death receptors 1–4 (DR receptors) wherein cell death is triggered by external signals [21]. Death receptors are typically expressed on cell membranes and facilitate the formation of multimolecular complexes through various adapter proteins and enzymes [22]. Several *stimuli* can enhance their transcription, indicating that the regulation of the extrinsic pathway is not solely dependent on the presence of death ligands [23]. Furthermore, apoptosis is also regulated by IAP (Inhibitor of Apoptosis) proteins, which exert their role by binding and inactivating the procaspases. To date, seven members of the IAP family have been identified, including survivin, which can bind and inhibit procaspases 3, 7, and 9, thus affecting both effector and initiator caspases. Modulators of IAP proteins, such as Smac/Diablo, negatively regulate these inhibitors, exhibiting pro-apoptotic properties [24] (Fig. 1). However, the regulation of apoptosis within a cell is influenced by members of the Bcl-2 family and also by the interplay of caspase inhibitors and their antagonists [25]. The concluding stage of apoptosis involves the generation of apoptotic bodies, wherein the cell disintegrates into numerous small vesicles recognizable by immune cells, particularly phagocytes and, more specifically, macrophages. This terminal phase is marked by the presentation of specific signals on the membrane, referred to as “eat me signals”, which include phagocytosis-inducing molecules like phosphatidylserine. Consequently, the apoptotic cell emits both membrane-bound and soluble signals that attract phagocytic cells, thereby promoting the effective clearance of apoptotic bodies [26].

### Role of integrins in anoikis

Cells sense their location through specific interactions between integrin family receptors and the ECM, where specific signals are recognized to prevent cell anoikis. Humans have 24 different integrins that are expressed on the cell membrane and are implicated in the recognition of ECM ligands [27]. Furthermore, integrins act as anchoring structures for adapter proteins and kinases involved in cell homeostasis maintenance [28].

It is well-known that integrins activate different signaling pathways according to the type of cell and the regulatory mechanisms in which they are involved. Four integrin types—α5β1, αvβ3, α1β1, and α6β1—are particularly rewarded for their contributions to cell survival across different cellular models, protecting against apoptosis and anoikis. The signaling molecules and pathways associated with integrins include pp125 Focal Adhesion Kinase (FAK), Src kinase, integrin-linked kinase, PI3K/Akt, and mitogen-activated protein kinase (MAPK) [6]. Moreover, several integrins bind to pp125-FAK, a non-receptor kinase that is activated upon cell adhesion, which engages with various signaling molecules, including Src, PI3-K, paxillin, and p130CAS, thereby facilitating the activation of multiple signaling pathways that govern cell migration, proliferation, and apoptosis. In addition, several integrins, such as α1β1, α5β1, and αvβ3, mediate the activation of Src, a member of the SRC kinase family, in the focal adhesion complex and determine ERK and PI3K/Akt signaling cascade. Thus, a Src early inactivation is responsible for a loss of cell adhesion, which turns out into anoikis induction [29].

Furthermore, integrins have the ability to either promote or inhibit actin-based structures, suggesting that the cytoskeleton also delineates the various pathways that connect integrins to the cytoskeletal scaffold. In particular, the cytoskeleton plays a key role in extending the integrin junction, enabling adhesive contacts to develop sufficient strength to survive the contractile forces associated with cellular movement and functionality. Alterations in these mechanical forces can modify the signaling pathways linked to adhesion, thus affecting cell survival [30]. Epithelial cell survival is also associated with the linkage between growth factors and adhesion mechanisms, and the lack of these conditions triggers the apoptotic pathway. A recent study reported that mammary epithelial cells depend on the adhesion of the ECM to laminin and, at the same time, to the insulin-like growth factor 1 (IGF-1) signal to guarantee correct tissue development [31, 32]. Moreover, increased levels of reactive oxygen species (ROS) lead to the activation of the tyrosine kinase Src by integrins that, as previously described, are involved in cell adhesion [33]. Further evidence highlighted the integrins and GFR crosstalk, ensuring target-dependent oligodendrocyte survival under growth factor-limiting conditions [34]. Finally, delineating the roles of integrins and growth factors in apoptosis regulation proved to be a complex attempt.

### Cell–cell interactions

As previously reported, anoikis mechanisms are not only related to cell–ECM interactions but also to cell-cell adhesion. Cell–cell interactions are facilitated by a group of membrane proteins known as cadherins, which establish either homotypic or heterotypic bonds between cells in a calcium-dependent manner [35, 36]. The disruption of these interactions can lead the anoikis activation. A recent study demonstrated that the adhesion mediated by E-cadherin activates the PI3K signaling pathway, which is essential for promoting cell survival. Moreover, cadherins may also play a role in cell survival through their indirect interactions with integrins. It has been noted that integrins, such as α2β1 and α3β, are often found at sites of cell–cell contact, where they can engage with EGF receptors, thereby activating downstream signaling pathways that inhibit apoptotic processes [6]. Additionally, recent investigations revealed that other molecules, including P- and L-selectin as well as NCAM, can initiate intracellular signaling cascades like those activated by ECM adhesion, involving pathways such as FAK, Src, PI3K/Akt, and MAPK [37].

## Targeting anoikis resistance in cancer

In physiological conditions, anoikis is an essential process for maintaining tissue homeostasis and preventing aberrant cell growth. Indeed, this programmed cell death acts as a safeguard to eliminate displaced cells, ensuring they do not colonize inappropriate locations and compromise tissue integrity. By eliminating detached or misplaced cells, anoikis assumes the role of a barrier to uncontrolled cell proliferation, tumor formation, and metastasis [38]. On the contrary, in cancer, cells acquire resistance to anoikis, enabling them to survive and proliferate in non-adherent conditions. AR represents a pivotal step in the metastatic cascade. During metastasis, cancer cells detach from the primary tumor, survive in suspension within the bloodstream or lymphatic system, and colonize distant tissues [39]. Therefore, tumor cells acquiring AR have enhanced invasive and migratory capacities. As a consequence, AR facilitates the dissemination process and promotes the establishment of secondary tumors [40, 41].

It is well known that the ability to evade anoikis is a hallmark of metastatic cancer and has been identified in numerous malignancies, including breast, lung, ovarian, pancreatic, and colorectal cancers (CRCs). It is a multifactorial process involving intrinsic genetic changes and extrinsic pathways [42, 43]. Integrins play a central role in mediating the interaction between cells and the ECM [44]. A key facilitator of this process is epithelial-to-mesenchymal transition (EMT), a phenotypic transformation that reduces cell adhesion and enhances mobility [33, 45]. EMT-associated transcription factors such as Snail, Twist, and ZEB1 suppress E-cadherin expression and activate survival pathways like PI3K/Akt, aiding in the evasion of anoikis [46]. Cancer cells often alter the expression of specific integrins, such as β1 and αvβ3, to promote survival under detachment conditions [47]. In addition to integrin signaling, AR is driven by the metabolic plasticity of cancer cells. Detached cells often undergo metabolic reprogramming, switching from oxidative phosphorylation to aerobic glycolysis (Warburg effect) to meet their energy demands in low-attachment states [48]. For example, in breast and pancreatic cancers, the detachment from ECM was linked to an increased reliance on autophagy and glycolysis, which provide energy and mitigate stress induced by ECM deprivation [49]. Autophagy, regulated by pathways such as PERK-AMPK, is activated in detached cells to promote survival by recycling intracellular components and reducing ROS production [50].

AR mechanisms vary across cancer types due to differences in tumor biology and microenvironmental factors [10]. In Table 1, a summary of recent studies on AR divided by cancer type is provided. The models used, as well as the mechanisms examined, are reported.

**Table 1:** Recent studies on the role of anoikis in cancer.

Mechanism of AR	Cancer type	Model used	Study type	Key findings	Ref.
Regulation of autophagy by PERK	Breast cancer	In vitro (MCF10A cells cultured in 2D and 3D)	preclinical	PERK activation upregulates autophagy and causes luminal filling during acinar morphogenesis by perpetuating a population of surviving autophagic luminal cells that resist anoikis.	[50]
Snail and EMT pathway upregulation	Breast cancer (triple-negative)	In vitro (MDA-MB-231 cells)	preclinical	Overexpression of MARCH2 suppresses tumor and phenocopies the effects of SNAIL downregulation and PTK6 inhibition	[54]
PI3K-Akt signaling pathway activation by TXA_2_-TP	Breast cancer	In vivo (mouse) in vitro (MDA-MB-231 and 4T1 cells)	preclinical	Among 19 phytochemicals apigenin, acts as anoikis sensitizer targeting TXA_2_-TP pathway	[80]
Caspase pathway	Lung carcinoma	In vitro (H460 and A549 cells) in vivo (Balb/c nude mice) ex vivo (commercial human tumor tissue array slides)	preclinical	Bit1 displays cancer suppressive function	[57]
PTEN/AKT signaling axis regulation by betaIII-tubulin	Non–small cell lung cancer	In vivo (mouse) in vitro (H460 and A549 cells)	preclinical	BetaIII-Tubulin blockage in vivo reduced tumor incidence and growth	[58]
Kinesin family member 5A (KIF5A) upregulation in cancer progression	Lung adenocarcinoma	In silico (GSEA) in vitro (BEAS-2B, NCI-H2087 and A549 cells)	preclinical	Ergotamine targets and inhibits the expression of KIF5A facilitating anoikis	[69]
ETM modulation by RHPN2 (cell proliferation and invasion)	Lung adenocarcinoma	In silico (GO and KEGG) in vitro (A549 and H1299 cells)	preclinical	Poorer prognosis in LUAD patients with elevated RHPN2 expression	[71]
Activation MEK/ERK pathway	Ovarian cancer	In vitro (SKOV-3 cells)	preclinical	Proliferation promotion E-caderin dependent and suppression of anoikis and maintenance of cellular survival	[52]
3D aggregate formation in ascitic fluids	Ovarian cancer	In vitro (OVCAR-4, A2780, OVCAR-8, IGROV-1, COV 362, OV-90 cells cultured in 3D as spheroids) ex vivo (human ascites samples)	clinical	αSMA and fibronectin are key components for the formation of 3D metastasizing units and plasticity maintenance necessary for implant and seeding into peritoneal lining. These 3D structures are protected from anoikis and chemotherapeutics	[59]
EGFR–AKT–BIM axis	Colorectal cancer	In vitro (HCT116 and SW620 cells) in vivo (Balb/c nude mice) human samples (80 normal 100 colon cancer tissues)	clinical	HCRP-1 regulates EGFR–AKT–BIM-mediated AR, promoting cancer metastasis.	[60]
high proliferative and CSC-like activity (to be clarified)	Colorectal cancer	In vitro (DLD-1 and SW620 cells) human tissues from 65 patients	clinical	KLF5 expression in cancer cells is an independent predictor of poor prognosis. KLF5 may be a novel therapeutic target	[61]
Bleb formation	Metastatic melanoma cancer	In vitro (MV3, M498 and A375 cells cultured in 2D and 3D)	preclinical	Bleb formation is associated with AR; inhibition of BRAF and MEK sensitizes to bleb and septin inhibition	[68]
TGFβ/TGFβRI/Smad2 pathway	Glioblastoma	In vitro (U87MG and U373 cells) in vivo (NMRI-nude mice) human tissues from 108 patients	clinical	CAV-1/α(5) β(1) integrins/TGFβRI are biomarkers of the tumor evolution/prognosis	[67]
ETM modulation and catalase and peroxidase activity	Hepatocellular carcinoma	In vitro (HCCLM3 cells) in vivo (Balb/c nude mice)	preclinical	A novel composite nanoenzyme based on mesoporous silica/nano-cerium oxide disrupts the EMT process	[81]

*AR* anoikis resistance, *CAV-1* caveolin-1, *CSC* Cancer stem-like cell, *EMT* epithelial–mesenchymal transition, *KLF5* Krüppel-like factor 5, *GO* Gene Ontology, *GSEA* Gene set enrichment analysis, *KEGG* Kyoto Encyclopedia of Genes and Genomes, *LUAD* Lung Adenocarcinoma, *TXA*_2_*-TP* thromboxane A2 receptor.

In breast cancer, the loss of E-cadherin induces AR by disrupting cell-cell adhesion and activating survival pathways [51]. Similar results were obtained for human ovarian cancer cells via the MEK/ERK pathway, which has been reported to help cells escape anoikis [52]. Integrins, such as β1 and α6, mediate anchorage-independent growth via FAK signaling [53]. EMT transcription factors like Snail and Twist are upregulated, while proteins such as HER2 and αB-crystallin promote survival in non-adherent states [33]. Triple-negative breast cancers often exhibit AR due to enhanced metabolic flexibility [54, 55].

Similarly, in lung cancer, proteins like caveolin-1 (CAV-1) and IL-13 receptor α2 contribute to AR by modulating autophagy and ROS responses. In addition, mitochondrial proteins like Bit1 and pathways such as PERK-mediated autophagy and IL13Rα2/PI3K/Akt signaling enhance AR, further driving metastatic potential [56–58]. In a study on ovarian cancer, cell aggregates in ascitic fluid from 15 patients use cell-cell adhesion and integrin signaling to evade anoikis. Moreover, αSMA and fibronectin are key components for the formation of 3D metastasizing units and plasticity maintenance necessary for implant and seeding into the peritoneal lining. These 3D structures are protected from anoikis and chemotherapeutics [59]. In CRC preclinical and clinical studies, tumor suppressors such as HCRP-1 [60] and transcription factors like KLF5 have been implicated in promoting survival under detachment conditions, suggesting their potential role as novel targets in CRC progression and metastatic cascade inhibition [10, 61, 62]. In pancreatic ductal adenocarcinoma (PDAC), AR is closely linked to the PI3K/Akt pathway and its downstream effectors. Overexpression of CDCP1 and the loss of tumor suppressors such as p16INK4a enhance survival and metastatic potential. The metabolic plasticity of PDAC cells, particularly the Warburg phenotype, also supports survival in non-adherent conditions [63].

AR is described in other cancer types, including hepatocellular carcinoma (HCC), esophageal squamous cell carcinoma (ESCC), and glioblastoma [64–66]. For instance, proteins like CAV-1, integrins α(5) β(1), and TGFβ/TGFβRI/Smad2 pathway in glioblastoma are critical for enabling survival and metastasis [67]. Malignant melanoma cells exhibit unique adaptations, including bleb formation, which functions as a survival hub during detachment [68].

Understanding the diversity of these mechanisms across cancer types is essential for developing targeted therapies. In fact, given its pivotal role in metastasis, targeting AR has emerged as an attractive field of study, which may lead to the discovery of new druggable targets [12, 69]. In several studies, most of them conducted employing various databases, Anoikis-triggered EMT genes and Anoikis-related genes (ARGs) were obtained and analyzed, and different signatures were developed and correlated with patients’ prognosis, therapeutic response, and chemoresistance acquisition [70, 71]. Indeed, Li et al., Tang et al., and Chen et al. identified five, ten, and seven ARG-prognostic signatures, respectively, in breast cancer by integrating Machine Learning, transcriptomic, clinical data, and experimental validation [72–74]. For this reason, therapeutic strategies targeting AR have shown promise in preclinical and early clinical studies. Agents that disrupt integrin signaling, such as cilengitide (targeting αvβ3 and αvβ5 integrins), can restore sensitivity to anoikis [75]. Other classes of anoikis inducer and sensitizer agents include FAK inhibitors. As an example, defactinib prevents the integrin-mediated activation of different downstream signal transduction pathways, including those involving RAS/MEK/ERK and PI3K/Akt. In this way, this inhibits tumor cell migration, proliferation, survival, and tumor angiogenesis [76]. In addition, drugs targeting EMT regulators, including Snail and Twist, are also considered a new therapeutic direction to overcome AR.

Another valuable field of investigation is the use of natural compounds as anoikis modulators [77]. As an example, curcumin, a polyphenol from turmeric, inhibits EMT and integrin-mediated survival pathways. In several studies it has been shown that resveratrol, a stilbenoid found in high concentrations in red grapes, among its pleiotropic functions, suppresses the PI3K/Akt pathway and inhibits cell growth, invasion, and proliferation by targeting NF-κB, Sirt1, Sirt3, LDH, PI-3K, mTOR, PKM2, R5P, G6PD, TKT, talin, and PGAM [78]. Flavonoids such as quercetin modulate ROS to sensitize cells to anoikis, and epigallocatechin gallate, a green tea polyphenol, reduces integrin expression to promote detachment-induced apoptosis [79]. Xu et al. screened 19 phytochemicals and identified apigenin as a potent anoikis sensitizer with anti-metastatic properties in both mouse and in vitro models of breast cancer [80]. Despite this preliminary evidence and fascinating data on the pro-anokis activity of natural compounds, the studies in this field meet several limitations, including a lack of biokinetic studies, poor bioavailability, and specificity. This emphasizes the need for innovative drug delivery systems or combination regimens to enhance efficacy. Nanomedicine, based on novel composite nanocarrier agents, may be considered another precious tool for treating AR. Among most recent studies Wang et al. developed a novel composite nano-enzyme based on mesoporous silica/nano-cerium oxide and obtained promising data for treating AR in HCC cells [81].

These novel approaches, both *stand-alone* or combined with standard treatment strategies, offer several advantages, including improved delivery, specificity, and a reduced toxicity profile; however, the assurance of their success passes through a deeper understanding of the tumor-specific determinants of AR. Moreover, the simultaneous modulation of multiple anoikis-related pathways, necessary to overcome the robust survival programs adopted by detached tumor cells, necessitates integrative analyses that combine high-throughput genomics, transcriptomics, and patient-derived data. With a deeper investigation of AR molecular machinery, in the future, we may significantly improve outcomes for patients with metastatic cancers, particularly those like GC where peritoneal metastasis leads to a very poor prognosis for patients.

## Prognostic value of anoikis in gastric cancer

GC represents malignant neoplasia of the gastrointestinal system characterized by heterogeneous features among the patients [82]. Despite the development of surgical procedures and techniques for diagnosis aimed at improving the patient’s quality of life, there is still a lack of GC diagnosis and treatment in clinics. Therefore, the identification of novel biomarkers, as well as the enhancement of the accuracy of this prediction for GC, is a crucial point. As previously reported, anoikis modulates the biological behaviors of several cancers, affecting cell viability and metastasis [6]. Understanding the role of anoikis is pivotal in cancer research, particularly in the dissemination and metastasis process. Cancer cells must overcome a critical challenge: surviving independently of their attachment to a substrate to successfully enter the circulatory system and disseminate. This requires the development of resistance to anoikis, which allows the cells to evade death upon detachment. Recently, the analysis of ARGs has been used as a novel prognostic strategy. Indeed, Li et al. identified SNCG as a specific ARG in GC by analyzing the Cancer Genome Atlas (TCGA) database. Particularly, they constructed a risk signature for GC patients to evaluate the role of ARGs in prognostic prediction to provide an original predictive tool for GC patients [83]. However, this study had some limitations, as both the gene expression analysis and the clinical GC cohort were derived from TCGA and the Gene Expression Omnibus repositories. Furthermore, these data should be validated by an in vitro*/*in vivo approach aimed at clarifying the SNGC role in GC progression.

Another study described the identification of further signatures of ARGs to predict the survival outcomes of GC patients, which includes PDK4, NOX4, SERPINE1, MMP11, DNMT1, EZH2, and SNCG genes. Specifically, GC patients were divided into high- and low-risk groups based on the ARGs identified, and the follow-up analyses disclosed that the high-risk score GC patients correlated with a poorer prognosis, which was confirmed by testing several cohorts [84]. The univariable and multivariable Cox analysis results displayed peculiar prediction values of the anoikis-related genes prognostic score within the high-score patients’ group, characterized by a worse overall survival. Further, by analyzing the immune landscape between high- and low-risk groups, the amount of immune cell infiltration was significantly increased in the high-risk group, a phenomenon probably related to the anoikis, as it could regulate cancer progression by affecting immune cell infiltration [84]. Although the authors performed a remarkable analysis of the public databases, the lack of in vitro*/*in vivo validation gives this study some limitations. Similarly, Cao et al. described another ARG signature constituted of nine genes, including CD36, MSLN, SERPINE1, EZH2, MMP11, OLFM3, TRAF2, TFDP1, and PDK4, whose overexpression is associated with a poor prognosis [85]. In this study, the ARG signature constructed is derived by analyzing public databases. Preliminary data confirm that the ARG signature is upregulated in GC cells; thus, it is necessary to corroborate these results using GC clinical specimens. In addition, another preliminary study identified a further ARG signature including 10 differentially expressed genes involved in both intracellular pathways, such as cell cycle, drug metabolism, and extrinsic pathways, including cGMP-PKG signaling, ECM, and receptor–ligand interaction [86]. Meng et al. described a further ARG signature based on lncRNA (AR-lncRNA) that is associated with GC prognosis and immune infiltration prediction [87]. This model included LINC02241, AL356417.2, AC012073.1, AL391152.1, PVT1, LINC01711, and CYMP-AS1 lncRNAs that are correlated with other tumor prognoses, including breast cancer, melanoma, HCC, glioblastoma, and GC [88–93]. However, as these studies have been performed only using an in silico approach, the validation by experimental research is crucial for deepening the AR-lncRNA role in GC progression. Another AR-lncRNAs-based GC prognostic model, constructed using retrospective data, includes AC091057.1, ADAMTS9.AS1, AC090825.1, AC084880.3, EMX2OS, HHIP.AS1, AC016583.2, EDIL3.DT, DIRC1, LINC01614, and AC103702.2, screened from AnoRGs-related lncRNAs [94]. According to other reports, most of the lncRNAs identified directly or indirectly affect tumor progression by regulating signaling pathways associated with cell proliferation and migration [95].

However, despite the convincing evidence provided in these reports, the lack of clinical validation of the above-mentioned prognostic biomarkers represents a crucial limitation.

## Mechanisms of anoikis resistance in gastric cancer

**Fig. 2:**
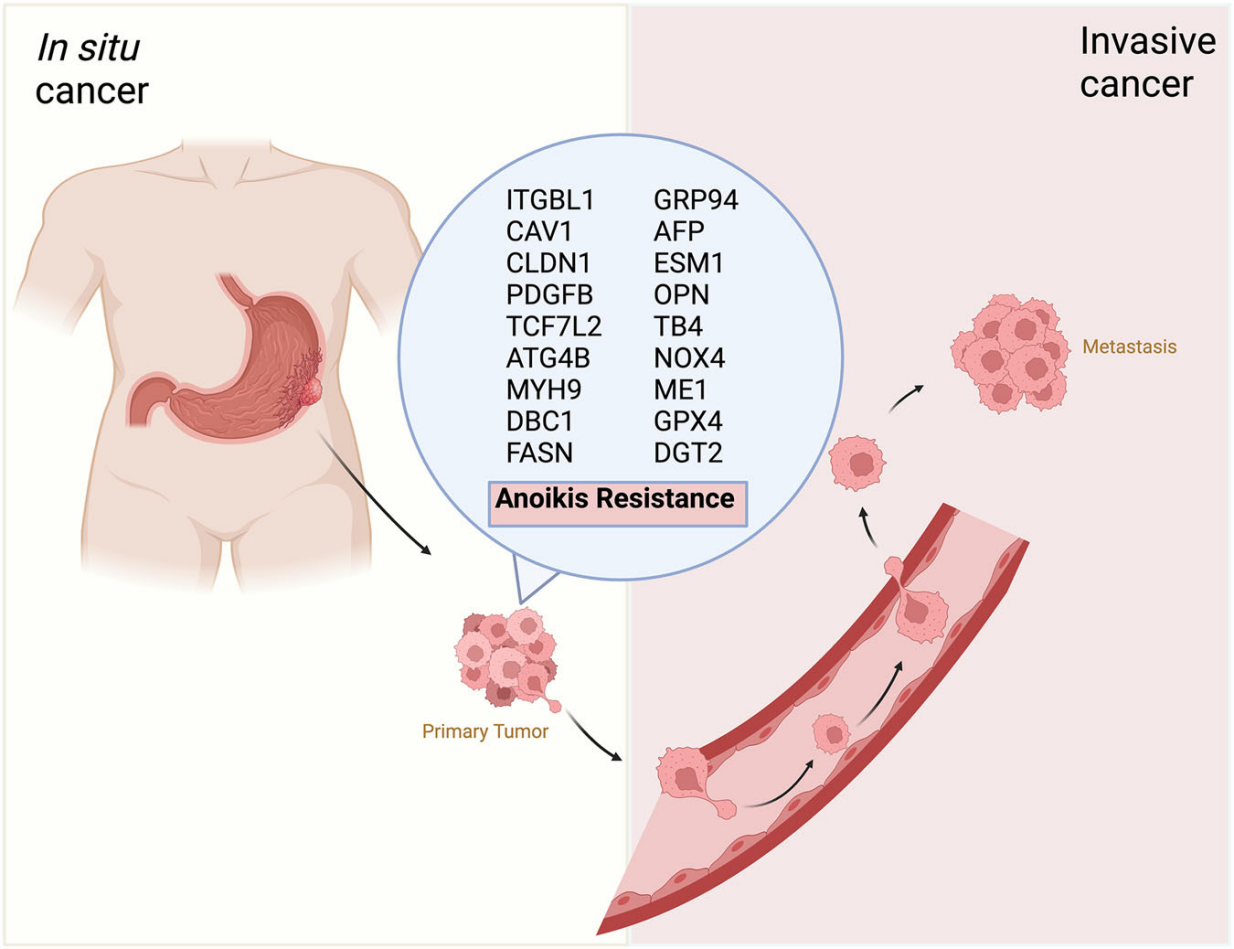
Overview of genes-inducing anoikis resistance in gastric cancer metastasis. Deregulation of Anoikis-Related Genes modulates transcriptional and metabolic pathways involved in cell detachment from ECM and promotes tumor dissemination.

The proliferation of tumor cells in GC is facilitated by their AR, which results in metastatic dissemination processes like contact loss, extravasation, invasion, and colonization of districts far from the primary sites typically associated with solid tumors (Fig. 2). In the early stages, cancer cells change their phenotype and cellular plasticity to survive. Growth factors, cytokines, and stress signals often cause the cell to activate survival pathways, leading to this change [96]. In GC, Integrin beta-like 1 (ITGBL1) via the Akt/Fibulin-2 axis (FBLN2) can enhance the metastatic capacity of the gastric tumor cell by determining AR. As evidenced both in vivo and in vitro, ITGB1 depletion leads to a decrease in AR, ultimately leading to metastatic spread [97]. Wang et al. have discovered how CAV1 regulates the process of avoiding death and growth-independent anchoring in EGFR-ITGB1 signaling. CAV1 is responsible for regulating AR by activating Src and pathways like PI3K/Akt and MEK/ERK [98]. A study on AGS cells revealed that a triterpenoid saponin called Platycodin D induces anoikis by activating stress-activated protein kinases like c-JUN and p38 MAPK [99]. Activating the Src and Akt signaling occurs also when claudin-1 (CLDN1) is upregulated. CLDN1 is a member of the tight junction family and is required for cell-cell adhesion. In GC, CLDN1 is overexpressed and acts as an anti-apoptotic protein by binding to β-catenin and promoting the survival of cells [100]. Furthermore, the tumor mass requires a higher supply of oxygen and nutrients and thus activates the process of new vessel formation, known as angiogenesis. The activation of angiogenesis in GC is linked to an increase in platelet-derived growth factor B (PDGFB) after a self-feedback loop from transcription factor C/EBPβ. Once released, PDGFB activates the MAPK signaling pathway in endothelial cells, resulting in a boost in metastatic progression and angiogenesis in GC cells [101].

Once cells detach and survive, they leave the primary site by activating chemotaxis, invasion, and migration processes. In GC, it was observed that the regulation of the plasminogen activating receptor, involved in the fibrinolytic process, is dependent on the transcription factor TCF7L2. Overexpression of TCF7L2 has been shown to promote AR by inhibiting the 3/7 caspase pathway in vivo [102]. A further transcription factor, transcription factor 2 (ATF2), promotes the transcription of growth differentiation factor 15 (GDF15). GDF15, also named the macrophage-1 inhibitor cytokine (MIC-1), is a secreted protein belonging to the superfamily of TGF-β. This increase in GDF15 transcription is associated with the activation of AR by the activation of protective autophagy [103]. Moreover, Wu et al. identified a novel pathway associated with autophagy and AR in GC, which involves ATG4B-*circ*SPECC1-ELAVL1. Particularly, being ATG4B, an RNA-binding protein overexpressed in GC detached cells, it undergoes ubiquitination and degradation upon interaction with *circ*SPECC1. However, as the *circ*SPECC1 is sequestered by the binding to ELAVL1, the inhibition of *circ*SPECC1-ELAVL1 linkage by the FDA-approved compound Lopinavir reestablishes *circ*SPECC1 abundance necessary for GC metastasis suppression [104]. Additionally, it has been demonstrated that nuclear factor MYH9, via its DNA-binding domain, also increases CTNNB1 transcription by inducing AR in both in vivo and in vitro studies. This crosstalk occurs due to the activation by CTNNB1 of the canonical pathway of Wnt/β-catenin, which increases gastric metastasis [105].

Inflammation is also a cellular mechanism that is associated with AR, in addition to protective autophagy. DBC1 is an overexpressed nuclear protein in GC and is responsible for reduced survival rates of patients with GC. The activation of the inflammatory pathway of NF-κB is controlled by this protein, which leads to cell AR [106]. Glucose-regulated protein 94 (GRP94) is also closely associated with cellular resistance to apoptosis, inflammation, proliferation, and invasion [107]. In GC, the transcription factor SP1 was found to induce overexpression of GRP94, which contributes to the onset of peritoneal metastases through the activation of YAP/TEAD1 and therefore of the Hippo pathway. Inhibition of YAP reduced the effects of GRP94 on AR and metastasis [108]. In addition, another mechanism implicated in the regulation of invasion and metastasis of AGS cells includes the α-fetoprotein, a pleiotropic tumor marker, which exerts its function by modulating the anoikis sensitivity, conferring resistance to cell death [109]. Further evidence identified the Endothelial cell-specific molecule 1 (ESM1), a secreted glycoprotein, as a novel biomarker negatively correlated with a poorer outcome in GC patients. Functionally, ESM1 expression promotes GC progression by regulating the EGFR/HER3-Akt/ANGPT2 pathway, which increases cell proliferation, migration/invasion and AR in GC cells [110]. Moreover, using a multi-omics approach in diffuse-type GC mice lacking E-cadherin, p53, and Smad4, Park et al. identified novel effectors such as osteopontin, which is involved in regulating EMT, and Tβ4, a potential E-cadherin repressor, which is involved in promoting cancer cell AR [111]. A summary of all the above-mentioned studies, including biomarkers and AR mechanisms and models, is reported in Table 2.

**Table 2:** Molecular factors related to anoikis resistance in GC.

Factor	Mechanism of AR	Model used	Ref.
ITGBL1	Akt-FBLN2 axis	in vitro (AGS and MKN45 cells) in vivo (mouse)	[97]
CAV-1	Activation of Src, PI3K/Akt, and MEK/ERK pathways	in vitro (SGC-7901 cells)	[98]
PD	Activation of c-JUN and p38MAPK	in vitro (AGS cells)	[99]
CLDN1	Activation of β-Catenin pathway	in vitro (AGS, NCI-N87, KATO III, HS746T and MKN45 cells) in vivo (mouse)	[100]
PDGFB	Activation of MAPK pathways	in vitro (MKN45) in vivo (mouse)	[101]
PLAUR	Inhibition of Caspases 3/7 mediated by PLAUR-induced transcription factor TCF7L2	in vitro (AGS, MGC-803 KATO III, HCG-27 and MKN45 cells) in vivo (mouse)	[102]
GDF15	Activation of the Protective Autophagy	in vitro (AGS and MKN45 cells)	[103]
ATG4B -*circ*SPECC1-ELAVL1	Regulation of Autophagy	in vitro (AGS, MKN45, MKN74 and MKN28, HCG-27, BGC823 and MGC803 in vivo (mouse)	[104]
MYH9	Activation of WNT/ β pathway upon CTNNB1 transcription	in vitro (MGC 80-3, MKN-45, HGC-27, AGS, NCI-N87, BGC-823, AGS, SNU-5, KATO III and SGC-7901) in vivo (mouse)	[105]
DBC1	NF-κB activation	in vitro (MKN45 and MGC803)	[106]
GRP94	Activation of YAP/TEAD1 and Hippo pathway	in vitro (MKN-45, MGC-803, HGC-27, NCI-N87 and GES-1) in vivo (mouse)	[107, 108]
AFP	E-Cadherin downregulation N-Cadherin upregulation	in vitro (AGS)	[109]
ESM1	Activation of EGFR/HER3-Akt/ANGPT2 pathway	in vitro (AGS, KATOIII, NCI-N87 and MKN45)	[110]
OPN/Tβ4	EMT regulation	in vitro (mouse primary gastric cells) in vivo (mouse)	[111]

*CAV-1* Caveolin 1, *CLDN1* Claudin-1, *DBC1* deleted in breast cancer 1, *EMT* Epithelial to Mesenchymal transition, *ESM1* Endothelial Cell Specific Molecule 1, *FBLN2* Fibulin-2, GDF15 Growth/ differentiation factor 15, *GRP94* Glucose Regulated Protein 94, *ITGBL1* Integrin subunit beta like 1, *PD* Platycodin D, *PDGFB* platelet-derived growth factor B, *PLAUR* Plasminogen Activator, Urokinase Receptor.

While the studies summarized in this section highlight a wide range of molecular players implicated in AR and GC, it is important to underline that most of these findings are based on in vitro models or animal studies. To date, there is a striking lack of validation in patient-derived systems or clinical studies. This limits the translational applicability of these biomarkers and mechanisms. A deeper mechanistic investigation, particularly within clinically relevant models, is urgently needed to confirm the role of these pathways in metastatic GC.

### Anoikis resistance and metabolism in gastric cancer

GC cells change their metabolism compared to their healthy counterparts. This “metabolic remodeling” (or metabolic reprogramming) is essential to supporting their rapid proliferation and survival in unfavorable conditions like hypoxia and a lack of nutrients in the tumor microenvironment [112, 113]. Activation of cell death programs like anoikis can result from changes in metabolic homeostasis, such as nutrient deficiency or ROS accumulation [10]. Conversely, tumor gastric cells possess a greater capacity to survive in metabolic stress conditions and develop mechanisms of AR. Upregulation of the NADPH oxidase 4 (NOX4) gene has been found to play a crucial role in the development of AR in GC cells. NOX4 is responsible for transporting electrons from NADPH to oxygen, which leads to the generation of H_2_O_2_ and ROS. An increase in protein levels causes an up-regulation of EGFR and a higher production of ROS, yielding higher AR in clustered cells than their adherent counterpart. In vivo, the inhibition of NOX4 led to a reduction in AR and metastatic progression of GC [114].

SMAD4 is another GC-related gene whose depletion is associated with an increase in ROS production. Particularly, SMAD4 downregulation correlates with the malic enzyme 2 (ME2) suppression. This latter is involved in lowering the oxidative stress by raising levels of NADPH. In the absence of ME2, the malic enzyme 1 (ME1) isoenzyme is upregulated by the ROS through the ME1-ETV4 axis, which results in resistance to death by anoikis and a rise in the invasion process [115].

In contrast to NOX4, Glutathione Peroxidase 4 (GPX4) is the enzyme responsible for preventing oxidative damage caused by iron accumulation. The gastric cell, via the intrinsic anoikis pathway, induces an increase in mitochondrial ROS production dependent on iron accumulation. As a result, the cell initiates the death process known as anoikis-induced ferroptosis. Sun et al. showed that the increase in GPX4 levels in cluster-detached cancer cells inhibited the ferroptosis process by reducing ROS levels, which promoted the migration of GC cells [116]. Adipose tissue and metabolism associated with fatty acids play a role in the metastasis and growth of gastric tumors related to AR. Obesity and/or a high-fat diet have been shown to increase lipid droplets in the peritoneum and the oxidation of fatty acids, as evidenced by multiple studies [117–119]. Diacylglycerol acyltransferase 2 (DGT2) deregulation was associated with C/EBPα-dependent increases in AR, leading to metastatic dissemination at the peritoneum level in the GC [117]. Fatty acid synthase (FASN) is another enzyme that contributes to AR, and it is involved in the synthesis of neonatal fat. In detail, the increase of FASN levels leads to AR through an increase in phosphorylation of ERK1/2 and activation of the anti-apoptotic protein Bcl-xL [120] (Table 3). In response to AR, several cancer types, including GC, experience an increase in proliferation, migration, and invasion processes. Even though comprehensive comparative studies are still lacking in GC, the evidence gathered so far suggests that metabolic reprogramming, along with integrin-mediated signaling and EMT, may constitute major mechanisms leading to AR in this malignancy. These processes converge to support survival under anchorage-independent conditions, maintain redox homeostasis, enhance migratory and invasive properties, and activate pro-survival pathways such as PI3K/Akt and MAPK/ERK [121]. Further investigations using multi- and pan-omics technologies, as well as advanced study models, are necessary to clarify this evidence, particularly in metastatic GC.

**Table 3:** Metabolic enzymes involved in the anoikis resistance in GC.

Factors	Mechanism of AR	Model used	Ref.
NOX4	Upregulation of EGFR Increasing ROS production	in vitro (GES-1, AGS and MKN45 cells) in vivo (mice) human tissue samples in silico (LinkedOmics)	[114]
ME1	Increasing ROS production by ME1-ETV4 axis,	in vitro (GES1, AGS, SGC7901, SNU216, BGC823, HGC27, MGC803, NUGC4, MKN45 and MKN74 cells) in silico (CCLE and TCGA) human tissue samples (*N* = 207) in vivo (BALB/c nude mice)	[115]
GPX4	Inhibition of anoikis-induced ferroptosis	in vitro (AGS, MKN45, HGC27 cells) tumor tissues from 54 GC patients with and without lymphatic metastasis	[116]
DGT2	Activation of C/EBPα	GC tissue specimens (*N* = 318) and freshly collected primary tumor tissues and matched metastasis tissues (*N* = 8) in vivo (BALB/c nude mice)	[117]
FASN	Activation of ERK1/2 pathways Activation of Bcl-xL	in vitro (GES-1, GC, MNK-45 and AGS cells)	[120]

*CCLE* Cancer Cell Line Encyclopedia, *FASN* Fatty Acid Synthase, *GPX4* Glutathione peroxidase 4, *ME1* Malic enzyme 1, *NOX4* NADPH Oxidase 4, *TCGA* The Cancer Genome Atlas.

## Conclusions and future perspectives

Anoikis has become a prominent topic in research, with a growing number of studies demonstrating the complex mechanisms cancer cells use to avoid this innate cell death, stressing the necessity for novel targeted approaches in developing effective therapies for anoikis-resistant tumors. Despite being a relatively recent area of study, intensive research on anoikis has yielded significant progress over a decade. In addition, from these investigations, AR appears to be a major driver of metastatic dissemination and therapeutic failure in GC. Through this review, we have examined the complex network of molecular mechanisms that allow GC cells to evade anoikis, including integrin-mediated signaling, EMT, autophagy, inflammation, and metabolic reprogramming. These adaptations enable detached tumor cells to survive under anchorage-independent conditions, promoting peritoneal dissemination and resistance to standard therapies. Nevertheless, our analysis has revealed that most of the current evidence on AR in GC derives from in vitro experiments or animal models. Few of the studies examined have employed patient-derived models or clinical specimens, revealing a significant translational gap in the field. Moreover, while multi-omics analyses have begun to identify prognostic ARG and lncRNA signatures, these have yet to be validated in large, well-annotated clinical cohorts.

To move the field forward, it is essential to integrate functional validation of druggable targets within patient-relevant models, such as patient-derived organoids, microfluidic systems, and xenografts. Moreover, investigating the crosstalk between AR and other regulated cell death mechanisms, such as ferroptosis, necroptosis, or pyroptosis, may uncover synergistic vulnerabilities. In the view of therapeutic applications, targeting AR could improve the efficacy of current regimens and reduce recurrence in metastatic GC. However, the development of such approaches will require a deeper mechanistic understanding of tumor- and context-specific AR pathways. Consolidating the current knowledge on AR in GC and validating the mechanisms, along with the identification of research gaps, remain critical points for translating these findings into effective, patient-tailored therapies.

## References

[1] Bray F, Laversanne M, Sung H, Ferlay J, Siegel RL, Soerjomataram I, et al. Global cancer statistics 2022: GLOBOCAN estimates of incidence and mortality worldwide for 36 cancers in 185 countries. CA Cancer J Clin. 2024;74:229–63. 38572751 10.3322/caac.21834

[2] Usui Y, Taniyama Y, Endo M, Koyanagi YN, Kasugai Y, Oze I, et al. *Helicobacter pylori*, homologous-recombination genes, and gastric cancer. N Engl J Med. 2023;388:1181–90. 36988593 10.1056/NEJMoa2211807

[3] Yang WJ, Zhao HP, Yu Y, Wang JH, Guo L, Liu JY, et al. Updates on global epidemiology, risk, and prognostic factors of gastric cancer. World J Gastroenterol. 2023;29:2452–68. 37179585 10.3748/wjg.v29.i16.2452 PMC10167900

[4] D’Amore T, Di Taranto, A, Berardi, G, Vita, V, Iammarino, M. Nitrate as food additives: reactivity, occurrence, and regulation. In: Nitrate handbook: environmental, agricultural and health effects. CRC Press: Florida; 2022. p 281–300.

[5] Guan WL, He Y, Xu RH. Gastric cancer treatment: recent progress and future perspectives. J Hematol Oncol. 2023;16:57. 37245017 10.1186/s13045-023-01451-3 PMC10225110

[6] Paoli P, Giannoni E, Chiarugi P. Anoikis molecular pathways and its role in cancer progression. Biochim Biophys Acta. 2013;1833:3481–98. 23830918 10.1016/j.bbamcr.2013.06.026

[7] Song P, Yakufujiang Y, Zhou J, Gu S, Wang W, Huo Z. Identification of important genes related to anoikis in acute myocardial infarction. J Cell Mol Med. 2024;28:e18264. 38526027 10.1111/jcmm.18264 PMC10962123

[8] Chakrabarti A, Bansal R, Mondal A, Upadhyay P, Gupta A, Verma P, et al. Epithelial homelessness: an atypical form of anoikis triggered by Leishmania interaction with epithelial cells. Future Microbiol. 2024;19:33–49. 37830931 10.2217/fmb-2023-0004

[9] Sakamoto S, Kyprianou N. Targeting anoikis resistance in prostate cancer metastasis. Mol Asp Med. 2010;31:205–14. 10.1016/j.mam.2010.02.001 PMC2988681 20153362

[10] Adeshakin FO, Adeshakin AO, Afolabi LO, Yan D, Zhang G, Wan X. Mechanisms for modulating anoikis resistance in cancer and the relevance of metabolic reprogramming. Front Oncol. 2021;11:626577. 33854965 10.3389/fonc.2021.626577 PMC8039382

[11] Foley JM, Scholten DJ 2nd, Monks NR, Cherba D, Monsma DJ, Davidson P, et al. Anoikis-resistant subpopulations of human osteosarcoma display significant chemoresistance and are sensitive to targeted epigenetic therapies predicted by expression profiling. J Transl Med. 2015;13:110. 25889105 10.1186/s12967-015-0466-4 PMC4419490

[12] Wang Y, Cheng S, Fleishman JS, Chen J, Tang H, Chen ZS, et al. Targeting anoikis resistance as a strategy for cancer therapy. Drug Resist Updat. 2024;75:101099. 38850692 10.1016/j.drup.2024.101099

[13] Pena-Blanco A, Garcia-Saez AJ. Bax, Bak and beyond—mitochondrial performance in apoptosis. FEBS J. 2018;285:416–31. 28755482 10.1111/febs.14186

[14] Solano-Galvez SG, Abadi-Chiriti J, Gutierrez-Velez L, Rodriguez-Puente E, Konstat-Korzenny E, Alvarez-Hernandez DA, et al. Apoptosis: activation and inhibition in health and disease. Med Sci. 2018;6:54. 10.3390/medsci6030054 PMC6163961 29973578

[15] Banjara S, Suraweera CD, Hinds MG, Kvansakul M. The Bcl-2 family: ancient origins, conserved structures, and divergent mechanisms. Biomolecules. 2020;10:128. 10.3390/biom10010128 PMC7022251 31940915

[16] Shibue T, Suzuki S, Okamoto H, Yoshida H, Ohba Y, Takaoka A, et al. Differential contribution of Puma and Noxa in dual regulation of p53-mediated apoptotic pathways. EMBO J. 2006;25:4952–62. 17024184 10.1038/sj.emboj.7601359 PMC1618103

[17] Bean GR, Ganesan YT, Dong Y, Takeda S, Liu H, Chan PM, et al. PUMA and BIM are required for oncogene inactivation-induced apoptosis. Sci Signal. 2013;6:ra20. 23532334 10.1126/scisignal.2003483 PMC3753585

[18] Yin XM. Bid, a critical mediator for apoptosis induced by the activation of Fas/TNF-R1 death receptors in hepatocytes. J Mol Med. 2000;78:203–11. 10933582 10.1007/s001090000099

[19] Gogvadze V, Robertson JD, Zhivotovsky B, Orrenius S. Cytochrome c release occurs via Ca2+-dependent and Ca2+-independent mechanisms that are regulated by Bax. J Biol Chem. 2001;276:19066–71. 11264286 10.1074/jbc.M100614200

[20] Reubold TF, Wohlgemuth S, Eschenburg S. Crystal structure of full-length Apaf-1: how the death signal is relayed in the mitochondrial pathway of apoptosis. Structure. 2011;19:1074–83. 21827944 10.1016/j.str.2011.05.013

[21] Ivanisenko NV, Seyrek K, Hillert-Richter LK, Konig C, Espe J, Bose K, et al. Regulation of extrinsic apoptotic signaling by c-FLIP: towards targeting cancer networks. Trends Cancer. 2022;8:190–209. 34973957 10.1016/j.trecan.2021.12.002

[22] Fu Q, Fu TM, Cruz AC, Sengupta P, Thomas SK, Wang S, et al. Structural basis and functional role of intramembrane trimerization of the Fas/CD95 death receptor. Mol Cell. 2016;61:602–13. 26853147 10.1016/j.molcel.2016.01.009 PMC4761300

[23] Gibson SB, Oyer R, Spalding AC, Anderson SM, Johnson GL. Increased expression of death receptors 4 and 5 synergizes the apoptosis response to combined treatment with etoposide and TRAIL. Mol Cell Biol. 2000;20:205–12. 10594023 10.1128/mcb.20.1.205-212.2000 PMC85076

[24] Silke J, Meier P. Inhibitor of apoptosis (IAP) proteins-modulators of cell death and inflammation. Cold Spring Harb Perspect Biol. 2013;5:a008730. 10.1101/cshperspect.a008730 PMC3552501 23378585

[25] Liu Z, Li H, Derouet M, Berezkin A, Sasazuki T, Shirasawa S, et al. Oncogenic Ras inhibits anoikis of intestinal epithelial cells by preventing the release of a mitochondrial pro-apoptotic protein Omi/HtrA2 into the cytoplasm. J Biol Chem. 2006;281:14738–47. 16461771 10.1074/jbc.M508664200

[26] Depraetere V. “Eat me” signals of apoptotic bodies. Nat Cell Biol. 2000;2:E104. 10854338 10.1038/35014098

[27] Kadry YA, Calderwood DA. Chapter 22: structural and signaling functions of integrins. Biochim Biophys Acta Biomembr. 2020;1862:183206. 31991120 10.1016/j.bbamem.2020.183206 PMC7063833

[28] Frisch SM, Screaton RA. Anoikis mechanisms. Curr Opin Cell Biol. 2001;13:555–62. 11544023 10.1016/s0955-0674(00)00251-9

[29] Diaz-Montero CM, Wygant JN, McIntyre BW. PI3-K/Akt-mediated anoikis resistance of human osteosarcoma cells requires Src activation. Eur J Cancer. 2006;42:1491–500. 16759849 10.1016/j.ejca.2006.03.007

[30] Delon I, Brown NH. Integrins and the actin cytoskeleton. Curr Opin Cell Biol. 2007;19:43–50. 17184985 10.1016/j.ceb.2006.12.013

[31] Rutherford TR, Elder AM, Lyons TR. Anoikis resistance in mammary epithelial cells is mediated by semaphorin 7a. Cell Death Dis. 2021;12:872. 34561423 10.1038/s41419-021-04133-5 PMC8463677

[32] Lee YJ, Streuli CH. Extracellular matrix selectively modulates the response of mammary epithelial cells to different soluble signaling ligands. J Biol Chem. 1999;274:22401–8. 10428812 10.1074/jbc.274.32.22401

[33] Dai Y, Zhang X, Ou Y, Zou L, Zhang D, Yang Q, et al. Anoikis resistance-protagonists of breast cancer cells survive and metastasize after ECM detachment. Cell Commun Signal. 2023;21:190. 37537585 10.1186/s12964-023-01183-4 PMC10399053

[34] O’Meara RW, Michalski JP, Kothary R. Integrin signaling in oligodendrocytes and its importance in CNS myelination. J Signal Transduct. 2011;2011:354091. 21637375 10.1155/2011/354091 PMC3101883

[35] Niessen CM, Leckband D, Yap AS. Tissue organization by cadherin adhesion molecules: dynamic molecular and cellular mechanisms of morphogenetic regulation. Physiol Rev. 2011;91:691–731. 21527735 10.1152/physrev.00004.2010 PMC3556819

[36] Kim SH, Li Z, Sacks DB. E-cadherin-mediated cell-cell attachment activates Cdc42. J Biol Chem. 2000;275:36999–7005. 10950951 10.1074/jbc.M003430200

[37] Yu X, Miyamoto S, Mekada E. Integrin alpha 2 beta 1-dependent EGF receptor activation at cell-cell contact sites. J Cell Sci. 2000;113:2139–47. 10825287 10.1242/jcs.113.12.2139

[38] Taddei ML, Giannoni E, Fiaschi T, Chiarugi P. Anoikis: an emerging hallmark in health and diseases. J Pathol. 2012;226:380–93. 21953325 10.1002/path.3000

[39] Drapela S, Gomes AP. Metabolic requirements of the metastatic cascade. Curr Opin Syst Biol. 2021;28:100381. 10.1016/j.coisb.2021.100381 PMC8535854 34693082

[40] Khan SU, Fatima K, Malik F. Understanding the cell survival mechanism of anoikis-resistant cancer cells during different steps of metastasis. Clin Exp Metastasis. 2022;39:715–26. 35829806 10.1007/s10585-022-10172-9

[41] Simpson CD, Anyiwe K, Schimmer AD. Anoikis resistance and tumor metastasis. Cancer Lett. 2008;272:177–85. 18579285 10.1016/j.canlet.2008.05.029

[42] Han YH, Wang Y, Lee SJ, Jin MH, Sun HN, Kwon T. Regulation of anoikis by extrinsic death receptor pathways. Cell Commun Signal. 2023;21:227. 37667281 10.1186/s12964-023-01247-5 PMC10478316

[43] Gundamaraju R, Lu W, Paul MK, Jha NK, Gupta PK, Ojha S, et al. Autophagy and EMT in cancer and metastasis: who controls whom?. Biochim Biophys Acta Mol Basis Dis. 2022;1868:166431. 35533903 10.1016/j.bbadis.2022.166431

[44] Liu F, Wu Q, Dong Z, Liu K. Integrins in cancer: emerging mechanisms and therapeutic opportunities. Pharm Ther. 2023;247:108458. 10.1016/j.pharmthera.2023.108458 37245545

[45] Pang X, He X, Qiu Z, Zhang H, Xie R, Liu Z, et al. Targeting integrin pathways: mechanisms and advances in therapy. Signal Transduct Target Ther. 2023;8:1. 36588107 10.1038/s41392-022-01259-6 PMC9805914

[46] Usman S, Waseem NH, Nguyen TKN, Mohsin S, Jamal A, Teh MT, et al. Vimentin is at the heart of epithelial mesenchymal transition (EMT) mediated metastasis. Cancers. 2021;13:4985. 10.3390/cancers13194985 PMC8507690 34638469

[47] Haake SM, Rios BL, Pozzi A, Zent R. Integrating integrins with the hallmarks of cancer. Matrix Biol. 2024;130:20–35. 38677444 10.1016/j.matbio.2024.04.003 PMC13078519

[48] Buchheit CL, Weigel KJ, Schafer ZT. Cancer cell survival during detachment from the ECM: multiple barriers to tumour progression. Nat Rev Cancer. 2014;14:632–41. 25098270 10.1038/nrc3789

[49] Pavlova NN, Zhu J, Thompson CB. The hallmarks of cancer metabolism: still emerging. Cell Metab. 2022;34:355–77. 35123658 10.1016/j.cmet.2022.01.007 PMC8891094

[50] Avivar-Valderas A, Bobrovnikova-Marjon E, Alan Diehl J, Bardeesy N, Debnath J, Aguirre-Ghiso JA. Regulation of autophagy during ECM detachment is linked to a selective inhibition of mTORC1 by PERK. Oncogene. 2013;32:4932–40. 23160380 10.1038/onc.2012.512 PMC3600386

[51] Bruner HC, Derksen PWB. Loss of E-cadherin-dependent cell-cell adhesion and the development and progression of cancer. Cold Spring Harb Perspect Biol. 2018;10:a029330. 10.1101/cshperspect.a029330 PMC5830899 28507022

[52] Dong LL, Liu L, Ma CH, Li JS, Du C, Xu S, et al. E-cadherin promotes proliferation of human ovarian cancer cells in vitro via activating MEK/ERK pathway. Acta Pharm Sin. 2012;33:817–22. 10.1038/aps.2012.30 PMC4010376 22543706

[53] Hamidi H, Ivaska J. Every step of the way: integrins in cancer progression and metastasis. Nat Rev Cancer. 2018;18:533–48. 30002479 10.1038/s41568-018-0038-z PMC6629548

[54] Ito K, Harada I, Martinez C, Sato K, Lee E, Port E, et al. MARCH2, a novel oncogene-regulated SNAIL E3 ligase, suppresses triple-negative breast cancer metastases. Cancer Res Commun. 2024;4:946–57. 38457262 10.1158/2767-9764.CRC-23-0090 PMC10977041

[55] Lanning NJ, Castle JP, Singh SJ, Leon AN, Tovar EA, Sanghera A, et al. Metabolic profiling of triple-negative breast cancer cells reveals metabolic vulnerabilities. Cancer Metab. 2017;5:6. 28852500 10.1186/s40170-017-0168-x PMC5568171

[56] Wang J, Luo Z, Lin L, Sui X, Yu L, Xu C, et al. Anoikis-associated lung cancer metastasis: mechanisms and therapies. Cancers. 2022;14:4791. 10.3390/cancers14194791 PMC9564242 36230714

[57] Yao X, Jennings S, Ireland SK, Pham T, Temple B, Davis M, et al. The anoikis effector Bit1 displays tumor suppressive function in lung cancer cells. PLoS ONE. 2014;9:e101564. 25003198 10.1371/journal.pone.0101564 PMC4086906

[58] McCarroll JA, Gan PP, Erlich RB, Liu M, Dwarte T, Sagnella SS, et al. TUBB3/betaIII-tubulin acts through the PTEN/AKT signaling axis to promote tumorigenesis and anoikis resistance in non-small cell lung cancer. Cancer Res. 2015;75:415–25. 25414139 10.1158/0008-5472.CAN-14-2740

[59] Capellero S, Erriquez J, Battistini C, Porporato R, Scotto G, Borella F, et al. Ovarian cancer cells in ascites form aggregates that display a hybrid epithelial-mesenchymal phenotype and allows survival and proliferation of metastasizing cells. Int J Mol Sci. 2022;23:833. 10.3390/ijms23020833 PMC8775835 35055018

[60] Chen F, Zhang L, Wu J, Huo F, Ren X, Zheng J, et al. HCRP-1 regulates EGFR-AKT-BIM-mediated anoikis resistance and serves as a prognostic marker in human colon cancer. Cell Death Dis. 2018;9:1176. 30518879 10.1038/s41419-018-1217-2 PMC6281589

[61] Takagi Y, Sakai N, Yoshitomi H, Furukawa K, Takayashiki T, Kuboki S, et al. High expression of Kruppel-like factor 5 is associated with poor prognosis in patients with colorectal cancer. Cancer Sci. 2020;111:2078–92. 32279400 10.1111/cas.14411 PMC7293098

[62] Pan YB, Xu WJ, Huang MS, Lu YD, Zhou YJ, Teng Y, et al. Anoikis-related signature identifies tumor microenvironment landscape and predicts prognosis and drug sensitivity in colorectal cancer. J Cancer. 2024;15:841–57. 38213716 10.7150/jca.91627 PMC10777033

[63] Mehra S, Deshpande N, Nagathihalli N. Targeting PI3K pathway in pancreatic ductal adenocarcinoma: rationale and progress. Cancers. 2021;13:4434. 10.3390/cancers13174434 PMC8430624 34503244

[64] Rahdan F, Abedi F, Dianat-Moghadam H, Sani MZ, Taghizadeh M, Alizadeh E. Autophagy-based therapy for hepatocellular carcinoma: from standard treatments to combination therapy, oncolytic virotherapy, and targeted nanomedicines. Clin Exp Med. 2024;25:13. 39621122 10.1007/s10238-024-01527-5 PMC11611955

[65] Chen L, Hu Y, Li Y, Zhang B, Wang J, Deng M, et al. Integrated multiomics analysis identified comprehensive crosstalk between diverse programmed cell death patterns and novel molecular subtypes in hepatocellular carcinoma. Sci Rep. 2024;14:27529. 39528670 10.1038/s41598-024-78911-4 PMC11555373

[66] Cao S, Li M, Cui Z, Li Y, Niu W, Zhu W, et al. Establishment and validation of the prognostic risk model based on the anoikis-related genes in esophageal squamous cell carcinoma. Ann Med. 2024;56:2418338. 39444152 10.1080/07853890.2024.2418338 PMC11504171

[67] Cosset EC, Godet J, Entz-Werle N, Guerin E, Guenot D, Froelich S, et al. Involvement of the TGFbeta pathway in the regulation of alpha5 beta1 integrins by caveolin-1 in human glioblastoma. Int J Cancer. 2012;131:601–11. 21901744 10.1002/ijc.26415

[68] Weems AD, Welf ES, Driscoll MK, Zhou FY, Mazloom-Farsibaf H, Chang BJ, et al. Blebs promote cell survival by assembling oncogenic signalling hubs. Nature. 2023;615:517–25. 36859545 10.1038/s41586-023-05758-6 PMC10881276

[69] Bao B, Yu X, Zheng W, Sun J. Ergotamine targets KIF5A to facilitate anoikis in lung adenocarcinoma. Clin Respir J. 2024;18:e70020. 39517115 10.1111/crj.70020 PMC11549061

[70] Liu Z, Zhang M, Cao X, Ma M, Han B. Anoikis-related gene signatures predict prognosis of lung adenocarcinoma patients and reveal immune infiltration. Transl Cancer Res. 2024;13:1861–75. 38737691 10.21037/tcr-23-2185 PMC11082686

[71] Yin L, Zhang Z, Yan Z, Yan Q. Multicenter cohort analysis of anoikis and EMT: implications for prognosis and therapy in lung adenocarcinoma. Discov Oncol. 2024;15:462. 39298078 10.1007/s12672-024-01293-6 PMC11413261

[72] Li L, Li L, Wang Y, Wu B, Guan Y, Chen Y, et al. Integration of machine learning and experimental validation to identify anoikis-related prognostic signature for predicting the breast cancer tumor microenvironment and treatment response. Genes. 2024;15:1458. 10.3390/genes15111458 PMC11594124 39596658

[73] Tang M, Rong Y, Li X, Pan H, Tao P, Wu Z, et al. Anoikis-related genes in breast cancer patients: reliable biomarker of prognosis. BMC Cancer. 2024;24:1163. 39300389 10.1186/s12885-024-12830-5 PMC11411761

[74] Chen C, Guo S, Chai W, Yang J, Yang Y, Chen G, et al. A comprehensive genome-based analysis identifies the anti-cancerous role of the anoikis-related gene ADH1A in modulating the pathogenesis of breast cancer. Mol Genet Genom. 2024;299:108. 10.1007/s00438-024-02200-y 39531174

[75] Girnius N, Henstridge AZ, Marks B, Yu JK, Gray GK, Sander C, et al. Cilengitide sensitivity is predicted by overall integrin expression in breast cancer. Breast Cancer Res. 2024;26:187. 39707454 10.1186/s13058-024-01942-2 PMC11660856

[76] Hanks SK, Ryzhova L, Shin NY, Brabek J. Focal adhesion kinase signaling activities and their implications in the control of cell survival and motility. Front Biosci. 2003;8:d982–96. 12700132 10.2741/1114

[77] Wang Y, Fleishman JS, Wang J, Chen J, Zhao L, Ding M. Pharmacologically inducing anoikis offers novel therapeutic opportunities in hepatocellular carcinoma. Biomed Pharmacother. 2024;176:116878. 38843588 10.1016/j.biopha.2024.116878

[78] Brockmueller A, Sameri S, Liskova A, Zhai K, Varghese E, Samuel SM, et al. Resveratrol’s anti-cancer effects through the modulation of tumor glucose metabolism. Cancers. 2021;13:188. 10.3390/cancers13020188 PMC7825813 33430318

[79] Ponte LGS, Pavan ICB, Mancini MCS, da Silva LGS, Morelli AP, Severino MB, et al. The hallmarks of flavonoids in cancer. Molecules. 2021;26:2029. 10.3390/molecules26072029 PMC8038160 33918290

[80] Xu RY, Zhang H, Li H, Chen W. Apigenin is an anoikis sensitizer with strong anti-metastatic properties in experimental breast cancer. Food Sci Hum Wellness. 2024;13:2221–33.

[81] Wang Y, Ding L, Feng J, Lin Z, Yao H, You X, et al. Mesoporous cerium oxide nanoenzyme for efficacious impeding tumor and metastasis via conferring resistance to anoikis. Biomaterials. 2025;314:122876. 39383776 10.1016/j.biomaterials.2024.122876

[82] Gao JP, Xu W, Liu WT, Yan M, Zhu ZG. Tumor heterogeneity of gastric cancer: from the perspective of tumor-initiating cell. World J Gastroenterol. 2018;24:2567–81. 29962814 10.3748/wjg.v24.i24.2567 PMC6021770

[83] Li Y, Pan Q, Cheng M, Wu Z. Identification and validation of anoikis-associated gene SNCG as a prognostic biomarker in gastric cancer. Aging. 2023;15:2541–53. 36996495 10.18632/aging.204626 PMC10120907

[84] Zhao Z, Li C, Peng Y, Liu R, Li Q. Construction of an original anoikis-related prognostic model closely related to immune infiltration in gastric cancer. Front Genet. 2022;13:1087201. 36685842 10.3389/fgene.2022.1087201 PMC9845267

[85] Cao J, Hong K, Cao Y, Cen K, Mai Y, Dai Y, et al. Development of anoikis-related genes signature to predict the prognosis in gastric cancer patients. Front Oncol. 2022;12:1096608. 36713571 10.3389/fonc.2022.1096608 PMC9878391

[86] Lin Y, Liu J. Anoikis-related genes as potential prognostic biomarkers in gastric cancer: a multilevel integrative analysis and predictive therapeutic value. IET Syst Biol. 2024;18:41–54. 38377622 10.1049/syb2.12088 PMC10996445

[87] Meng WJ, Guo JM, Huang L, Zhang YY, Zhu YT, Tang LS, et al. Anoikis-related long non-coding RNA signatures to predict prognosis and immune infiltration of gastric cancer. Bioengineering. 2024;11:893. 10.3390/bioengineering11090893 PMC11428253 39329635

[88] Li Z, Li Y, Wang X, Yang Q. Identification of a six-immune-related long non-coding RNA Signature for predicting survival and immune infiltrating status in breast cancer. Front Genet. 2020;11:680. 32733537 10.3389/fgene.2020.00680 PMC7358358

[89] Zhang M, Yang L, Wang Y, Zuo Y, Chen D, Guo X. Comprehensive prediction of immune microenvironment and hot and cold tumor differentiation in cutaneous melanoma based on necroptosis-related lncRNA. Sci Rep. 2023;13:7299. 37147395 10.1038/s41598-023-34238-0 PMC10163022

[90] Zhu L, Zhang XP, Xu S, Hu MG, Zhao ZM, Zhao GD, et al. Identification of a CD4+ conventional T cells-related lncRNAs signature associated with hepatocellular carcinoma prognosis, therapy, and tumor microenvironment. Front Immunol. 2022;13:1111246. 36700197 10.3389/fimmu.2022.1111246 PMC9868629

[91] Huang L, Wang Z, Liao C, Zhao Z, Gao H, Huang R, et al. PVT1 promotes proliferation and macrophage immunosuppressive polarization through STAT1 and CX3CL1 regulation in glioblastoma multiforme. CNS Neurosci Ther. 2024;30:e14566. 38287522 10.1111/cns.14566 PMC10805395

[92] Wang Y, Chen X, Jiang F, Shen Y, Fang F, Li Q, et al. A prognostic signature of pyroptosis-related lncRNAs verified in gastric cancer samples to predict the immunotherapy and chemotherapy drug sensitivity. Front Genet. 2022;13:939439. 36147488 10.3389/fgene.2022.939439 PMC9485603

[93] Yuan M, Jia Y, Xing Y, Wang Y, Liu Y, Liu X, et al. Screening and validation of platelet activation-related lncRNAs as potential biomarkers for prognosis and immunotherapy in gastric cancer patients. Front Genet. 2022;13:965033. 36186426 10.3389/fgene.2022.965033 PMC9515443

[94] Lu L, Yu M, Huang W, Chen H, Jiang G, Li G. Construction of stomach adenocarcinoma prognostic signature based on anoikis-related lncRNAs and clinical significance. Libyan J Med. 2023;18:2220153. 37300839 10.1080/19932820.2023.2220153 PMC10259301

[95] Chen S, Gu J, Zhang Q, Hu Y, Ge Y. Development of biomarker signatures associated with anoikis to predict prognosis in endometrial carcinoma patients. J Oncol. 2021;2021:3375297. 34992654 10.1155/2021/3375297 PMC8727165

[96] Li W, Ng JM, Wong CC, Ng EKW, Yu J. Molecular alterations of cancer cell and tumour microenvironment in metastatic gastric cancer. Oncogene. 2018;37:4903–20. 29795331 10.1038/s41388-018-0341-x PMC6127089

[97] Shen K, Xia W, Wang K, Li J, Xu W, Liu H, et al. ITGBL1 promotes anoikis resistance and metastasis in human gastric cancer via the AKT/FBLN2 axis. J Cell Mol Med. 2024;28:e18113. 38332530 10.1111/jcmm.18113 PMC10853594

[98] Wang K, Zhu X, Mei D, Ding Z. Caveolin-1 contributes to anoikis resistance in human gastric cancer SGC-7901 cells via regulating Src-dependent EGFR-ITGB1 signaling. J Biochem Mol Toxicol. 2018;32:e22202. 30088837 10.1002/jbt.22202

[99] Chun J, Joo EJ, Kang M, Kim YS. Platycodin D induces anoikis and caspase-mediated apoptosis via p38 MAPK in AGS human gastric cancer cells. J Cell Biochem. 2013;114:456–70. 22961809 10.1002/jcb.24386

[100] Huang J, Zhang L, He C, Qu Y, Li J, Zhang J, et al. Claudin-1 enhances tumor proliferation and metastasis by regulating cell anoikis in gastric cancer. Oncotarget. 2015;6:1652–65. 25544763 10.18632/oncotarget.2936 PMC4359322

[101] Du S, Yang Z, Lu X, Yousuf S, Zhao M, Li W, et al. Anoikis resistant gastric cancer cells promote angiogenesis and peritoneal metastasis through C/EBPbeta-mediated PDGFB autocrine and paracrine signaling. Oncogene. 2021;40:5764–79. 34341514 10.1038/s41388-021-01988-y

[102] Zhang T, Wang B, Su F, Gu B, Xiang L, Gao L, et al. TCF7L2 promotes anoikis resistance and metastasis of gastric cancer by transcriptionally activating PLAUR. Int J Biol Sci. 2022;18:4560–77. 35864968 10.7150/ijbs.69933 PMC9295057

[103] Gao X, Zhang Z, Li Q, Tai G, Wang Z. GDF15 enhances anoikis resistance and metastasis of gastric cancer through protective autophagy. Cell Signal. 2024;124:111457. 39389179 10.1016/j.cellsig.2024.111457

[104] Wu Y, Chen Y, Yan X, Dai X, Liao Y, Yuan J, et al. Lopinavir enhances anoikis by remodeling autophagy in a circRNA-dependent manner. Autophagy. 2024;20:1651–72. 38433354 10.1080/15548627.2024.2325304 PMC11210930

[105] Ye G, Yang Q, Lei X, Zhu X, Li F, He J, et al. Nuclear MYH9-induced CTNNB1 transcription, targeted by staurosporin, promotes gastric cancer cell anoikis resistance and metastasis. Theranostics. 2020;10:7545–60. 32685004 10.7150/thno.46001 PMC7359096

[106] Huan Y, Wu D, Zhou D, Sun B, Li G. DBC1 promotes anoikis resistance of gastric cancer cells by regulating NF-kappaB activity. Oncol Rep. 2015;34:843–9. 26035299 10.3892/or.2015.4007

[107] Pugh KW, Alnaed M, Brackett CM, Blagg BSJ. The biology and inhibition of glucose-regulated protein 94/gp96. Med Res Rev. 2022;42:2007–24. 35861260 10.1002/med.21915 PMC10003671

[108] Shi Q, Lu Y, Du Y, Yang R, Guan Y, Yan R, et al. GRP94 promotes anoikis resistance and peritoneal metastasis through YAP/TEAD1 pathway in gastric cancer. iScience. 2024;27:110638. 39252968 10.1016/j.isci.2024.110638 PMC11381759

[109] Lu S, Ma Y, Sun T, Ren R, Zhang X, Ma W. Expression of alpha-fetoprotein in gastric cancer AGS cells contributes to invasion and metastasis by influencing anoikis sensitivity. Oncol Rep. 2016;35:2984–90. 26986949 10.3892/or.2016.4678

[110] Yang YC, Ho KH, Pan KF, Hua KT, Tung MC, Ku CC, et al. ESM1 facilitates the EGFR/HER3-triggered epithelial-to-mesenchymal transition and progression of gastric cancer via modulating interplay between Akt and angiopoietin-2 signaling. Int J Biol Sci. 2024;20:4819–37. 39309430 10.7150/ijbs.100276 PMC11414391

[111] Park JW, Kim MS, Voon DC, Kim SJ, Bae J, Mun DG, et al. Multi-omics analysis identifies pathways and genes involved in diffuse-type gastric carcinogenesis induced by E-cadherin, p53, and Smad4 loss in mice. Mol Carcinog. 2018;57:947–54. 29528141 10.1002/mc.22803

[112] Tufail M, Jiang CH, Li N. Altered metabolism in cancer: insights into energy pathways and therapeutic targets. Mol Cancer. 2024;23:203. 39294640 10.1186/s12943-024-02119-3 PMC11409553

[113] Addeo M, Di Paola G, Verma HK, Laurino S, Russi S, Zoppoli P, et al. Gastric cancer stem cells: a glimpse on metabolic reprogramming. Front Oncol. 2021;11:698394. 34249759 10.3389/fonc.2021.698394 PMC8262334

[114] Du S, Miao J, Zhu Z, Xu E, Shi L, Ai S, et al. NADPH oxidase 4 regulates anoikis resistance of gastric cancer cells through the generation of reactive oxygen species and the induction of EGFR. Cell Death Dis. 2018;9:948. 30237423 10.1038/s41419-018-0953-7 PMC6148243

[115] Lu YX, Ju HQ, Liu ZX, Chen DL, Wang Y, Zhao Q, et al. ME1 regulates NADPH homeostasis to promote gastric cancer growth and metastasis. Cancer Res. 2018;78:1972–85. 29654155 10.1158/0008-5472.CAN-17-3155

[116] Sun J, Li J, Pantopoulos K, Liu Y, He Y, Kang W, et al. The clustering status of detached gastric cancer cells inhibits anoikis-induced ferroptosis to promote metastatic colonization. Cancer Cell Int. 2024;24:77. 38369484 10.1186/s12935-024-03260-1 PMC10874580

[117] Li S, Wu T, Lu YX, Wang JX, Yu FH, Yang MZ, et al. Obesity promotes gastric cancer metastasis via diacylglycerol acyltransferase 2-dependent lipid droplets accumulation and redox homeostasis. Redox Biol. 2020;36:101596. 32506038 10.1016/j.redox.2020.101596 PMC7276427

[118] Lo Iacono M, Modica C, Porcelli G, Brancato OR, Muratore G, Bianca P, et al. Targeting of the peritumoral adipose tissue microenvironment as an innovative antitumor therapeutic strategy. Biomolecules. 2022;12:702. 10.3390/biom12050702 PMC9138344 35625629

[119] Mitchelson KAJ, O’Connell F, O’Sullivan J, Roche HM. Obesity, dietary fats, and gastrointestinal cancer risk-potential mechanisms relating to lipid metabolism and inflammation. Metabolites. 2024;14:42. 10.3390/metabo14010042 PMC10821017 38248845

[120] Yu L, Wang X, Du Y, Zhang X, Ling Y. FASN knockdown inhibited anoikis resistance of gastric cancer cells via P-ERK1/2/Bcl-xL pathway. Gastroenterol Res Pract. 2021;2021:6674204. 34456997 10.1155/2021/6674204 PMC8390150

[121] He C, He J. Metabolic reprogramming and signaling adaptations in anoikis resistance: mechanisms and therapeutic targets. Mol Cell Biochem. 2025;480:3315–3342. 10.1007/s11010-024-05199-3 39821582

